# Vitreoretinal biomarkers of retinopathy of prematurity using handheld optical coherence tomography: a review

**DOI:** 10.3389/fped.2023.1191174

**Published:** 2023-05-31

**Authors:** Deepika Kubsad, Masis A. Ohan, Jolan G. Wu, Michelle T. Cabrera

**Affiliations:** ^1^ University of Washington School of Medicine, Seattle, WA, United States; ^2^ Department of Ophthalmology, University of Washington, Seattle, WA, United States; ^3^ Division of Ophthalmology, Seattle Children's Hospital, Seattle, WA, United States

**Keywords:** retinopathy of prematurity (ROP), handheld optical coherence tomography, vitreoretinal biomarkers, vison screening, preterm infant, childhood blindness, plus disease

## Abstract

Retinopathy of prematurity (ROP) is caused by abnormal retinal vascularization in premature infants that has the potential for severe long-term vision impairment. Recent advancements in handheld optical coherence tomography (OCT) have enabled noninvasive, high-resolution, cross-sectional imaging of the infant eye at the bedside. The use of handheld OCT devices in the diagnosis of ROP in premature infants has furthered our understanding of disease state and progression. This review discusses the known and novel biomarkers of ROP severity in premature infants identified through handheld OCT and potential for future directions.

## Introduction

Retinopathy of prematurity (ROP) is a vascular proliferative disorder characterized by peripheral retinal neovascularization. It remains the leading cause of preventable blindness worldwide, affecting about 32,000 infants each year (1). The International Classification of Retinopathy of Prematurity outlines the classification of ROP based on zone or location of disease, stage, or the degree of peripheral extraretinal neovascularization, and plus disease or the presence of increased dilation and tortuosity of posterior retinal blood vessels (2). Currently, there are two standard options for the treatment of ROP: laser retinal photocoagulation and intravitreal anti-vascular endothelial growth factor (VEGF) therapy (2). Although ROP often regresses spontaneously, severe cases of ROP can cause retinal detachment leading to significant lifelong blindness. Timely identification and treatment of infants with advanced ROP remains crucial for improved outcomes.

The current guidelines from the American Academy of Ophthalmology and the American Academy of Pediatrics recommend a retinal screening examination for infants with a birth weight of ≤1500 g or gestational age of ≤30 weeks due to risk for ROP (3). Bedside examination with binocular indirect ophthalmoscopy remains the gold standard for ROP screening (3). Wide-field retinal fundus photography is considered an acceptable alternative (4). The use of binocular indirect ophthalmoscopy (BIO) and wide-field retinal photography both require ocular contact with topical anesthetic, eye manipulation, and bright light exposure. All of these factors have been identified as significant stressors for premature infants undergoing ROP screening (5–7). Although noncontact retinal fundus imaging may be less stressful than BIO, the use of bright light is still a risk factor for cardiorespiratory distress (8). Fluorescein angiography has been demonstrated to provide useful information regarding vascularization and possible leakage in advanced cases, although it is not as commonly employed in clinical settings (9).

**Figure 1:**
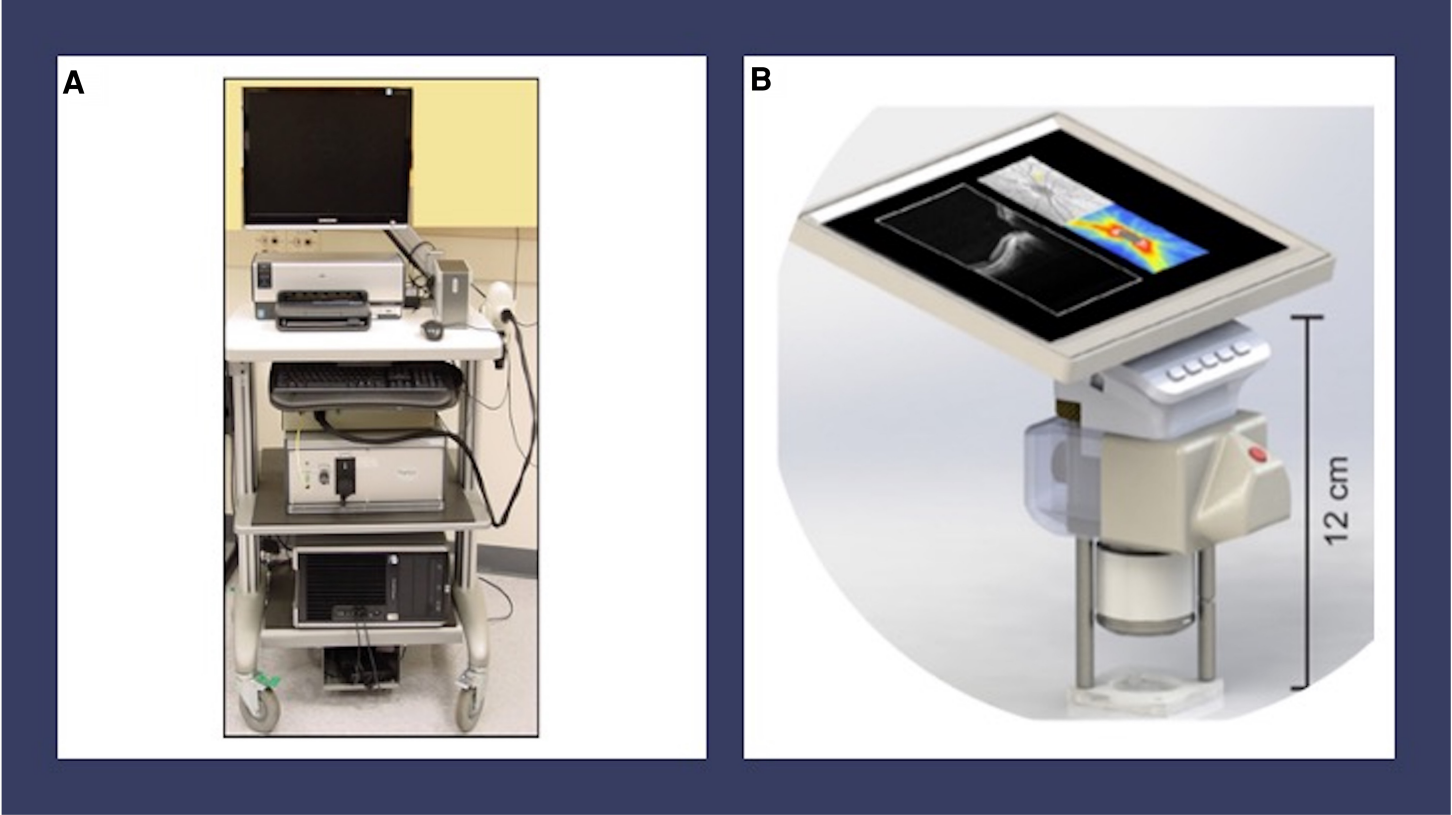
Handheld OCT devices. (**A**) Envisu C2300 handheld spectral domain optical coherence tomography (Leica Microsystems, Germany). (**B**) Model for investigational handheld swept source optical coherence tomography from the University of Washington Department of Bioengineering. This device has a handheld screen which features live video of the pupil and B-scan OCT images.

The use of handheld noncontact, near-infrared OCT imaging is a promising imaging modality as it does not use significant visible light, eyelid speculum or scleral depression in most cases (10). In fact, Mangalesh et al. demonstrated that the use of handheld OCT is significantly less invasive than that of BIO based on its impact on vital signs and other indicators of preterm infant stress (11). Handheld OCT provides the further advantage of high-resolution non-invasive capture of the microanatomy of the retinal vasculature and other vitreoretinal morphology. Previously, the use of tabletop OCT was limited to patients over the age of 3 due to ergonomic reasons. In 2010, handheld spectral domain OCT (SD-OCT) was developed and optimized for infant use, utilizing modifications to the reference arm, focus, and scan settings to address unique and dynamic characteristics of biometric infant ocular features (10). This technology allowed for noncontact, portable imaging of awake supine premature infants at the bedside (10). Images captured using handheld SD-OCT from premature infants with ROP have demonstrated previously unseen structural features of prematurity including cystoid macular edema (CME), epiretinal membranes (ERM), retinal blood vessel characteristics consistent with plus disease, punctate hyperreflective vitreous opacities, vitreous bands, subclinical retinal detachment and retinoschisis (12–17). The Envisu C2300 SD-OCT became the first commercially available handheld OCT approved by the Food and Drug Administration in 2012 (Leica Microsystems, Germany) (18) (Figure 1A). This was followed by a stand-mounted iVue system from Optovue Inc. with a removable 2.2 kg scanner (19). Rapid acquisition speeds in both devices made it easier to capture ocular images from pediatric populations. Another device called Heidelberg Spectralis Flex Module (Heidelberg Engineering, Heidelberg, Germany) incorporated the acquisition lens in a mobile arm that could be moved up to 100 cm from the device's main body (20). Despite these advancements, these instruments are susceptible to motion artifact in awake infants and have a steep learning curve for the user.

The development of an investigational swept source OCT (SS-OCT) from the University of Washington Department of Bioengineering provided a faster imaging speed of 200 kHz and a ranging distance of up to 12 mm, allowing for faster visualization of the anatomic region of interest in premature infants (21, 22) (Figure 1B). Key innovations, including a handheld screen featuring both live video of the pupil and B-scan OCT images, improved the usability of this device over older models (21, 22). Other centers have developed similar technologies for awake infant retinal imaging using handheld SS-OCT (23–25). Handheld OCT angiography (OCTA) is a more recent development in OCT imaging technology, which allows for the detailed visualization of the retinal vasculature without intravenous injections, although this technology is challenged by movement artifact in the awake infant population (21–24, 26). Finally, wide-field handheld OCT improves the characterization of peripheral findings in ROP, although it does require ocular contact (23, 25).

Handheld OCT is advantageous for retinal imaging in ROP due to its largely noninvasive methodology with decreased stress levels for infants. Handheld OCT has the potential to uncover important biomarkers associated with ROP. This paper summarizes known potential handheld OCT ROP severity biomarkers. In the future, combining these biomarkers into models for ROP risk identification may lead to a non-invasive ROP diagnostic screening tool.

## Vitreous biomarkers

**Figure 2:**
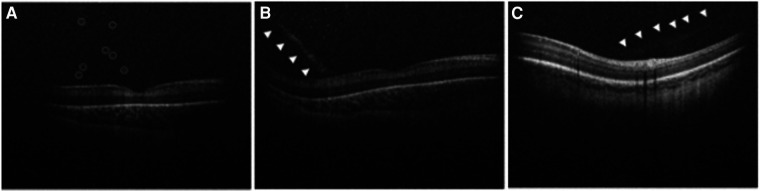
Handheld spectral domain optical coherence tomography images obtained from premature infants showing vitreous biomarkers of retinopathy of prematurity. (**A**) Numerous punctate hyperreflective vitreous opacities (circled) hovering above the retina are demonstrated in a premature infant with Stage 3 retinopathy of prematurity. (**B**) A tractional vitreous band (arrowheads) approaches at a steep angle, making contact with the retina near the fovea. Punctate hyperreflective vitreous opacities above the retina are also shown in a premature infant with Stage 3 retinopathy of prematurity. (**C**) Another example of a vitreous band (arrowheads) in a premature infant with Stage 0 retinopathy of prematurity.

Handheld OCT is an excellent modality to capture punctate hyperreflective vitreous opacities and vitreous bands, both new biomarkers for disease. Premature infants may experience loss of clarity of the vitreous at the macroscopic or microscopic level due to subtle forms of persistent fetal vasculature, associated vitreous hemorrhage, inflammation, hemorrhage, or protein accumulation (12, 27). Punctate hyperreflective vitreous opacities seen by handheld SD-OCT (Figure 2A) were first identified in premature infants by Zepeda et al. (17). Legocki et al. went on to document that 61 of 92 (66%) premature infants being screened for ROP harbored punctate hyperreflective vitreous opacities visualized by handheld SD-OCT imaging (12). That study also identified an association between the presence of punctate hyperreflective vitreous opacities and ROP severity based on stage, plus disease, and Type I ROP diagnosed from binocular indirect ophthalmoscopy. The significance of punctate hyperreflective vitreous opacities in premature infants who are at risk of ROP remains uncertain. However, it is speculated that these opacities could represent blood, protein, white blood cells, or retinal astrocyte precursors that are shed from the ROP ridge in advanced ROP (12). In cases of persistent fetal vasculature, the opacities may represent blood or other vascular remnants (12, 28).

Semi-automated quantification of punctate hyperreflective vitreous opacities identified an association between opacity density and recent ROP laser treatment, supporting an inflammatory etiology for these findings (12). Opacity density also increased with ROP severity based on stage at particular time points, suggesting that this finding could be used as an ROP severity biomarker in real time (12). Scoville et al. went on to identify punctate hyperreflective vitreous opacities in 25 of 28 (89%) healthy full-term newborns, similar to 41 of 50 (82%) premature infants, using an investigational handheld SS-OCT device from the University of Washington (28). That study identified a much higher density of vitreous opacities in premature infants compared to full term newborns (28). This newer device confirmed an association between punctate hyperreflective vitreous opacity density and clinical ROP severity based on zone and stage (28). Associations with intraventricular hemorrhage and subchorionic hemorrhage were also seen (28). Altogether, these findings point to the importance of punctate hyperreflective vitreous opacities as biomarkers of ROP.

Vitreous material shadowing was first described by Lee et al. (15) with the use of handheld SD-OCT, however the source of the shadowing was not described. These vitreous bands were later characterized by Zepeda et al. as linear opacities that appeared denser than the surrounding vitreous (Figures 2B,C) (17). That study described the presence of vitreous bands in 24 of 65 (37%) premature infants screened for ROP (17). Vitreous bands were categorized as tractional or non-tractional (17). Tractional bands made contact with the retinal surface, and non-tractional bands hovered parallel to the retinal surface (17). Legocki et al. went on to demonstrate that the presence of tractional bands was associated with a diagnosis of plus disease, suggesting that tractional bands are a marker for advanced ROP (12). A proposed theory suggests that the vitreous bands may arise from a vitreoschisis cavity that acts as a framework for retinal glial and inflammatory cells to migrate, resulting in the formation of non-tractional bands (17). These bands may represent tractional vectors in the vitreous targeted during vitrectomy for stage 4 ROP (29). Zepeda et al. (17) also found an association between the development of vitreous bands and the presence of CME and ERM, pointing to a tractional etiology for these mysterious features of the premature infant macula, as previously theorized by Vinekar et al. (30).

## Cystoid macular edema

CME is a common finding in premature infants undergoing screening for ROP, with prevalence ranging from 29% to 60% and unknown etiology (30–35). However, the association between CME and ROP has been variable. Some studies have suggested that CME may be a risk factor for the development of ROP or may be a sign of more severe ROP (31, 32). Other studies have not found a significant association between CME and ROP stage (30, 33–35). It is important to note that the pathogenesis of CME is unknown in this population and may have multiple causes, including inflammation, normal developmental changes, the influence of VEGF and vitreoretinal traction (17, 34). The resolution of CME is reported in most cases but not in all (30, 32, 33).

## Foveal development biomarkers

**Figure 3:**
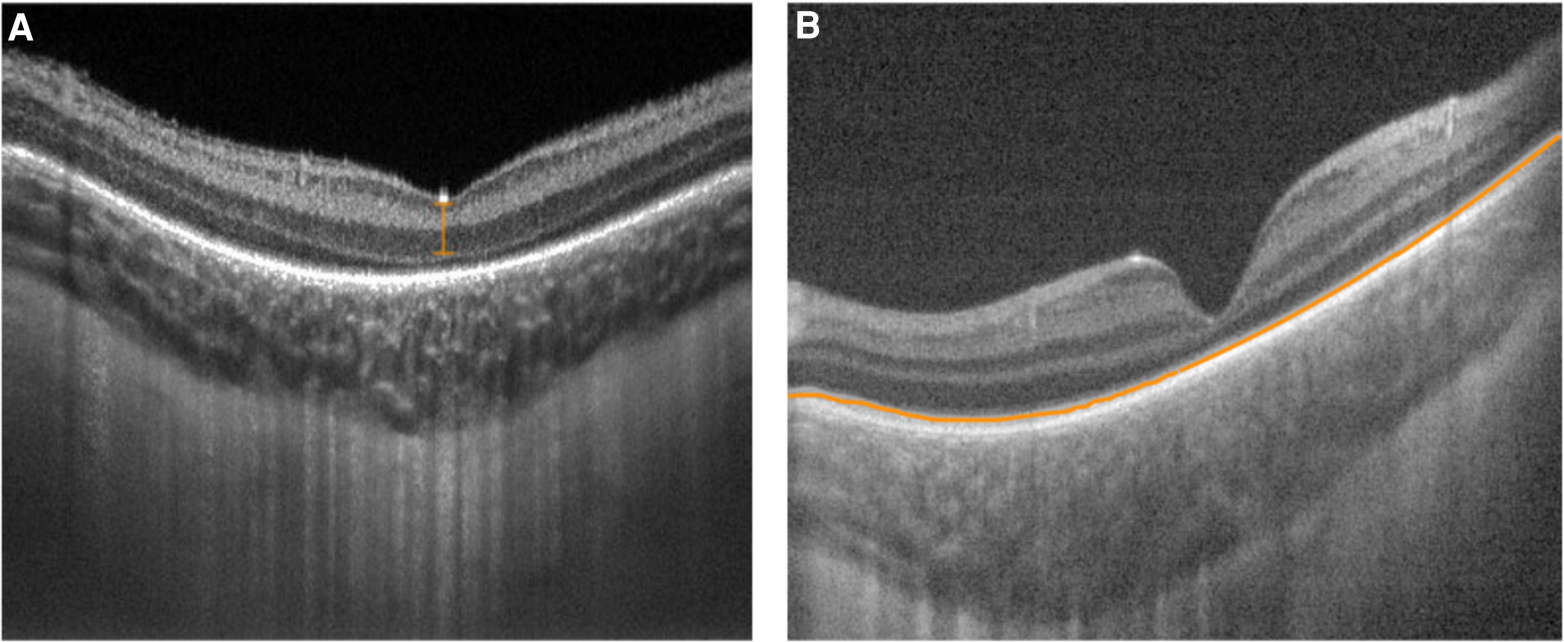
Handheld swept source optical coherence tomography images with variable foveal maturity. (**A**) Persistent inner retinal layers (orange bracket) and the absence of an ellipsoid zone with a relatively thin choroid at the fovea in the right eye of a premature infant (birth weight 629 g; gestational age 23 5/7 weeks) imaged at postmenstrual age of 36 5/7 weeks. (**B**) Normal inner retinal layers with an ellipsoid zone (orange line) present with a relatively thick choroid at the fovea in the left eye of a full term infant (birth weight 3509 g; gestational age 36 weeks) imaged at postmenstrual age of 36 1/7 weeks.

Structurally, the adult fovea contains inner and outer retinal layers that facilitate high-acuity central vision. During development, inner retinal layers undergo centrifugal displacement from the foveal center (36, 37). The outer retina contains highly packed retinal cones that make up a progressively thickening outer nuclear layer (36, 37). Although the fovea does not reach maturity until two years of age, inner retinal differentiation mostly occurs before full-term birth, while outer retinal differentiation mostly takes place after birth (36, 37). Handheld SD-OCT imaging of premature infants screened for ROP revealed shallow fovea, increased foveal thickness, and persistence of inner retinal layers with many infants continuing to exhibit foveal maturation over time after birth (38, 39). Vajzovic et al. used a handheld SD-OCT to correlate premature infant foveal pit morphology, including inner segment/outer segment development, to findings on histologic specimens (40). O'Sullivan et al. and Lawson et al. used handheld SS-OCT to identify that extremely premature infants had persistent shallow foveal pits in which the inner retinal layer (IRL) failed to migrate outward from the foveal center (41, 42) (Figure 3A). Univariate analysis revealed that advanced ROP and ROP requiring treatment were risk factors for lower maximum parafovea/foveal retinal thickness ratio, a measure of foveal immaturity, although multivariate analysis pointed to gestational age as the most important factor predicting persistent foveal immaturity (41). Furthermore, Anwar et al. found that ROP was associated with an increased foveal width in premature infants imaged with handheld SD-OCT (43).

In general, this research implies that the degree of prematurity may interfere with the normal development of a mature fovea by disrupting the process of retinal remodeling (41). Because foveal maturity may closely correspond with peripheral retinal maturity and retinal vascular development, these changes may therefore serve as biomarkers for ROP risk.

## Choroidal features

The metabolic demands of the developing retina are among the highest in the human body (44). The choroid is the primary blood supply for the photoreceptors and may serve an important role in outer retinal development (44). Any alteration in choroidal growth during maturation may affect retinal maturity or vitreoretinal pathologic abnormalities (44). Choroidal development continues after birth for preterm infants with increases in choroidal thickness detected over time using handheld SD-OCT (45). Although preterm infants maintained an upward trajectory in choroidal thickness, they lagged behind their term counterparts (42, 45) (Figure 3). Huang et al. identified a relationship between choroidal thickness and outer retinal maturity based on the presence of an ellipsoid zone in full term newborns imaged using handheld SS-OCT (46). Lawson et al. identified a similar relationship between foveal and parafoveal choroidal thickness and outer retinal maturity in premature infants imaged with handheld SS-OCT, while also detecting increasing choroidal thickness with increasing inner retinal maturity based on fewer persistent inner retinal layers (42).

**Figure 4:**
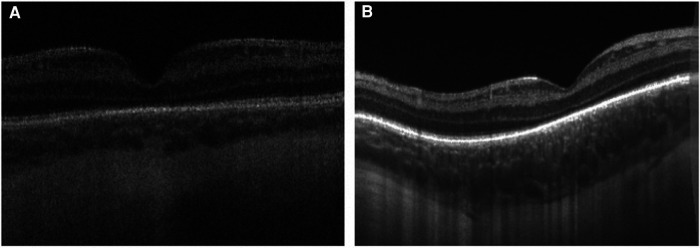
Handheld swept source optical coherence tomography images obtained from premature infants demonstrated that thinner choroid is associated with higher retinopathy of prematurity stage, worse plus disease, lower gestational age, and lower birth weight. (**A**) Swept source optical coherence tomography of a premature infant with Stage 2 retinopathy of prematurity, without plus disease and (**B**) Another infant with Stage 0 retinopathy of prematurity and no plus disease demonstrates thicker choroid.

Thinner choroid among premature infants was associated with higher ROP stage and plus disease, as well as lower gestational age, and lower birth weight (31, 34) (Figure 4). Although the etiology of the relationship between choroidal thickness and ROP severity remains unclear, animal models have demonstrated that deficient vascularity in the choroid in ROP, possibly due to oxidative damage, can lead to choroidal involution and subsequent photoreceptor loss (47). Furthermore, choroidal and retinal development appear to be linked, which may contribute to a relationship between choroidal development and ROP severity (42, 46). Choroidal thickness, therefore, may serve as an additional ROP severity biomarker during the newborn period.

## Dome-shaped macula

**Figure 5:**
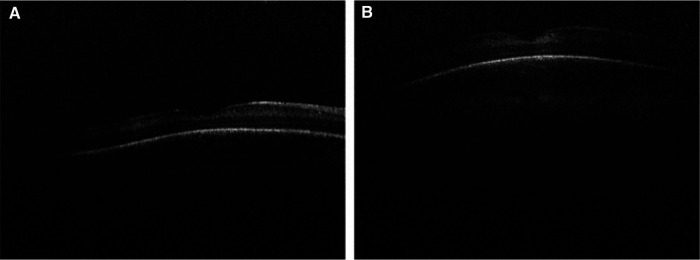
Handheld spectral domain optical coherence tomography images of dome-shaped maculae in premature infants. Dome-shaped macula was seen in 24 of 37 (65%) premature infants undergoing retinopathy of prematurity screening. Their presence was associated with low birth weight, plus disease, and a diagnosis of retinopathy of prematurity. **(A)** An example of subtle dome-shaped macula in a premature infant (birth weight 1210 g; gestational age 28 6/7 weeks) imaged at postmenstrual age of 35 1/7 weeks. **(B)** An example of more prominent dome-shaped macula seen in a premature infant (birth weight 499 g; gestational age 27 1/7 weeks) imaged at postmenstrual age of 40 weeks.

Dome-shaped macula, a convex configuration of the chorioretinal contour noted on OCT, has been extensively studied in adults and is known to be strongly associated with high myopia (48). There have been rare cases noted among children and adolescents (49, 50). Legocki et al. described these findings in 24 of 37 (65%) premature infants imaged using handheld SD-OCT while undergoing screening for ROP (51) (Figure 5). In this study, dome-shaped macula exhibited an elevation that was consistently found at the fovea and detected in 33 out of 37 eyes (89%) over a prolonged period of time, suggesting that this finding represents a true anatomic feature rather than imaging artifact related to probe orientation (51). This study failed to measure the dome height for most study subjects due to limitations of the device in achieving accurate measurements, therefore it is unknown whether the degree of elevation in dome-shaped macula has any clinical impact (51). Scoville et al. went on to report the findings of dome-shaped macula in 6 of 28 (21%) preterm infants and 1 of 50 (2%) full term infants based on handheld SS-OCT (28). The etiology of dome-shaped macula in the preterm population remains elusive, but it may represent a sign of immature ocular morphology, a potential risk factor for ROP. Some proposed theories mention that the presence of dome-shaped macula may be a phase of normal macular development due to mismatched premature retina and scleral size (51). Alternatively, the pressure gradient between intraocular pressure and posterior pressure may affect the contour of the retinal pigment epithelium and Bruch's membrane, leading to dome-shaped macula (51). Among premature infants imaged using handheld SD-OCT, the presence of dome-shaped macula was associated with low birth weight, plus disease and a diagnosis of ROP, suggesting that it may serve as an ROP severity biomarker (51).

## OCT features of plus disease

**Figure 6:**
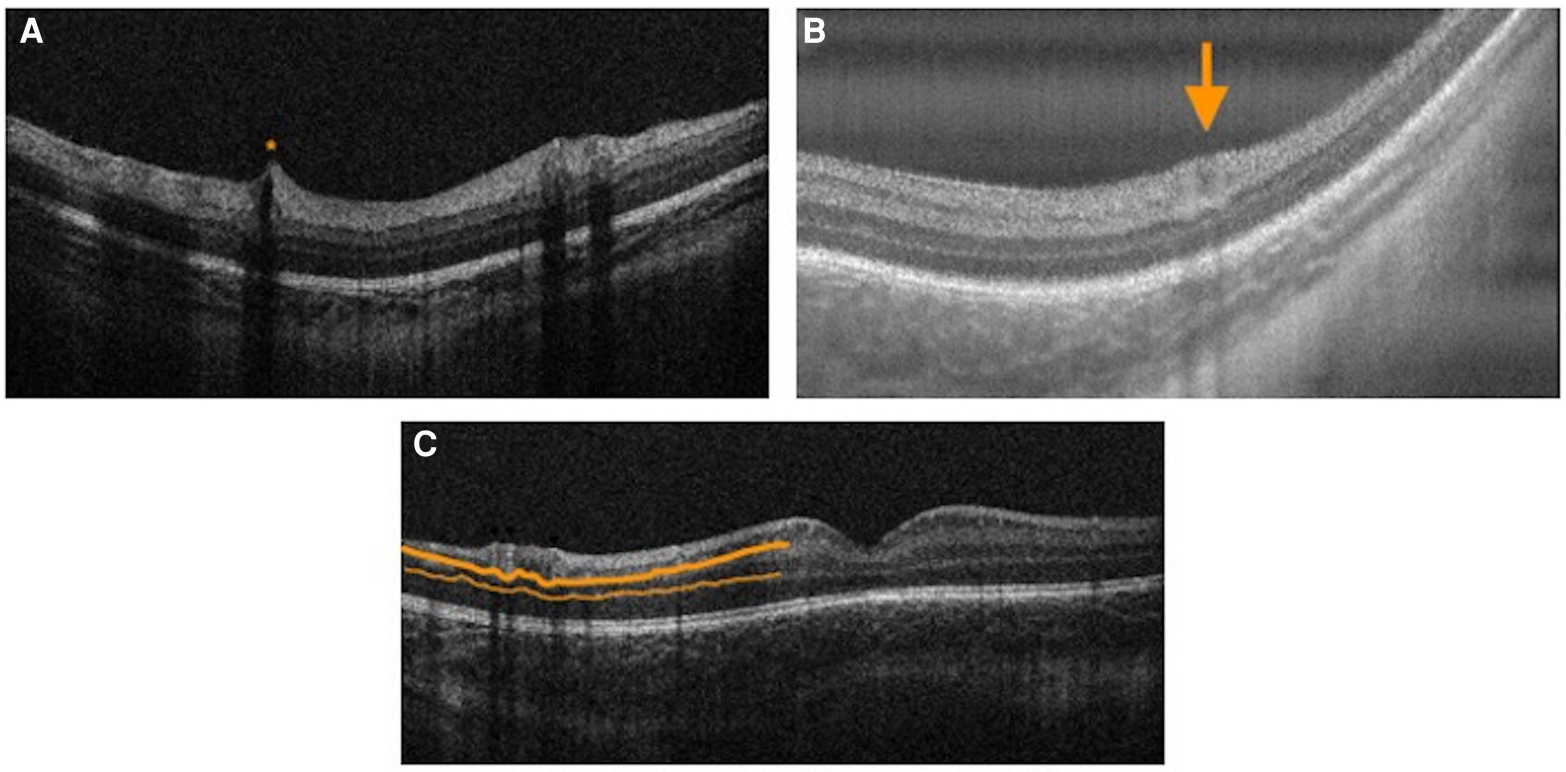
Vascular abnormality score on OCT features. (**A**) Handheld spectral domain optical coherence tomography image demonstrating hyporeflective vessels and vessel elevation (orange asterisk) in the right eye of a premature infant with zone III, Stage 3 retinopathy of prematurity with pre-plus disease (birth weight 1310 g, gestational age 31 2/7 weeks) imaged at postmenstrual age of 41 1/7 weeks. (**B**) Handheld swept source optical coherence tomography image demonstrating retinal space (orange arrow) in the right eye of a premature infant with zone II, stage 0 retinopathy of prematurity with plus disease (birth weight 1098 g; gestational age 30 2/7 weeks) imaged at postmenstrual age of 34 2/7 weeks. (**C**) Handheld spectral domain optical coherence tomography image demonstrating scalloped retinal layers (thick orange line — inner plexiform layer; thin orange line — outer plexiform layer) in the right eye of a premature infant with zone III, stage 3 retinopathy of prematurity with plus disease (birth weight 1310 g, gestational age 31 2/7 weeks) imaged at postmenstrual age of 46 1/7 weeks.

Plus disease is a form of ROP characterized by retinal vessel dilation and tortuosity at the posterior pole (52). The presence of plus disease is an important indication for treatment in ROP (53). Currently, subjective bedside indirect ophthalmoscopy remains the gold standard for the diagnosis of plus disease (3). Maldonado et al. identified cross-sectional retinal features on handheld SD-OCT that appear to strongly correlate with clinical plus disease based on indirect ophthalmoscopy (16). Based on these features, they proposed a Vascular Abnormality Score by OCT (VASO) consisting of vessel elevation, scalloped retinal layers, hypo-reflective vessels and retinal spaces (16). The VASO score appears to have the potential to diagnose clinical plus disease using handheld OCT, especially in infants <37 weeks postmenstrual age (16) (Figure 6). More recently, Seely et al. used ROPtool and handheld SS-OCT to generate retinal vessel maps for detecting plus or pre-plus disease with high reliability and accuracy (54). The combination of OCT images and objective grading of vessel morphology using software is promising to establish future diagnostic utility of OCT in a clinical setting.

## OCT angiography

**Figure 7:**
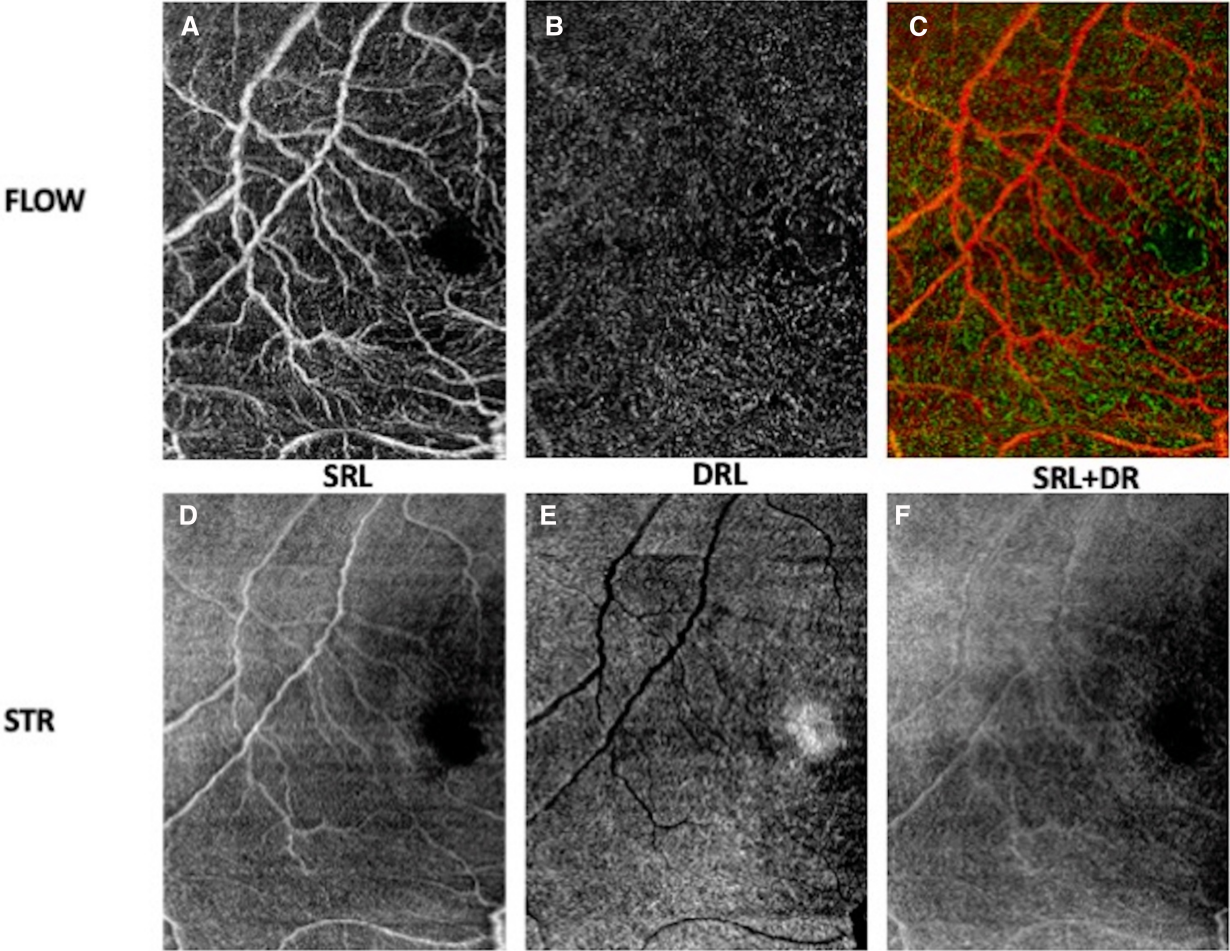
OCT angiogram demonstrating the foveal avascular zone in the right eye of a premature infant with zone II, stage 0 retinopathy of prematurity (birth weight 636 g; gestational age 27 0/7 weeks) imaged at postmenstrual age 36 0/7 weeks. En face OCT flow images **(A–C)** and en face OCT structural images **(D–F)** of superficial retinal layer (SRL), deep retinal layer (DRL) and superficial and deep retinal layers.

Optical coherence tomography angiography (OCTA) is a noninvasive, dye-less imaging modality that allows for visualization of the superficial and deep retinal capillary plexus and choroidal circulation (55). The recent advancements in acquisition speed have expanded its use for preterm infants (21–24, 26). In 2014, Hsu et al. quantified the foveal avascular zone (FAZ) and identified retinal vascular variation in sedated infants based on age, race, and axial length (56) (Figure 7). Kothari et al. went on to further identify smaller FAZs associated with thicker inner retinal layers among low-birth-weight infants with treatment warranted ROP (57). Zhou et al. was the first to use SS-OCTA to quantify the retinal vasculature morphology based on vessel area density, nonperfusion area, vessel diameter, and vessel tortuosity parameters from OCTA images in the awake preterm and full term infant population (26). They identified increased tortuosity in the preterm group compared to full term newborns (26). That study also found that vessel area density of preterm infants with advanced ROP was higher than that of those without advanced ROP (26). Vinekar et al. went on to confirm these findings; they identified that the superficial capillary plexus vessel density was lower in preterms born without ROP, with type 1 ROP, and with type 2 ROP compared to that of the term group (58). The FAZ in the superficial and deep capillary plexus was significantly smaller in preterms compared to that of controls as well as among preterms <31 weeks gestational age compared to those >31 weeks (58). In another report, the use of OCTA in a preterm neonate with aggressive posterior ROP was used to track regression of flat neovascularization at the border of peripherally laser-treated retina (59). As the utilization and feasibility of OCTA imaging increases, it has the potential to serve as an ROP screening tool.

## Peripheral OCT

**Figure 8:**
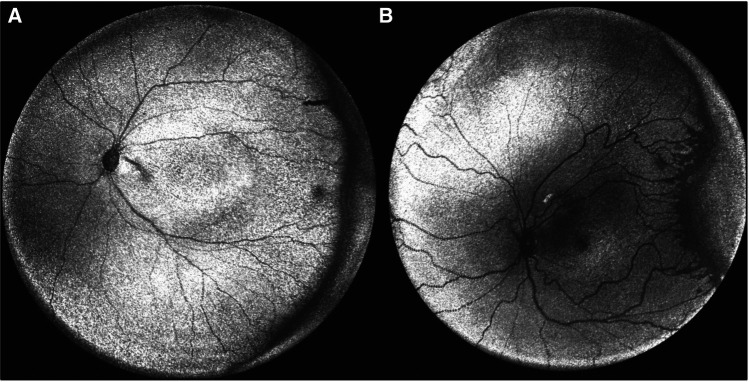
Peripheral optical coherence tomography retinal (OCT) images. (**A**) En face volumetric ultra-wide field OCT image of a premature infant with zone II, mild stage 2 retinopathy of prematurity (birth weight 690 g; gestational age 24 1/7 weeks) and (**B**) Another premature infant with zone I, stage 2 with popcorn neovascularization retinopathy of prematurity (birth weight 622 g; gestational age 24 1/7 weeks). (Courtesy of Peter Campbell, MD and Yifan Jian, PhD).

The current standards in determination of zone, stage, and plus disease in ROP are qualitative and often subject to variability among clinicians (60). Stage of ROP could be measured more objectively using OCT; however, peripheral features of ROP are often difficult to access with OCT due to the limited field of view. Although several groups have demonstrated that increasing field of view is possible, the assessment of peripheral ROP staging remains challenging due to the tradeoff between image quality and field of view (23, 61). Chen et al. achieved peripheral imaging of Zone I or posterior Zone II ROP using non-contact handheld SD-OCT (Bioptigen, now Leica) in select cases, correlating these findings to histopathology (61). This work demonstrated that Stage 1 ROP is associated with tapering from three retinal layers to one (61). In Stage 2, pronounced thickening is visible (61). Stage 3 is characterized by preretinal buds and bands (61). Nguyen et al. were able to obtain a field of view of 105° using an ultra-wide field OCT scan (Figure 8) and went on to identify an association between ridge thickness and ROP stage (62). Scruggs et al. used wide-field OCT (>55°) in conjunction with scleral depression to successfully document the peripheral ROP stage (63). A more recent study from the same group achieved more rapid acquisition time using wide-field (55°) SS-OCTA to document 3 examples of effective peripheral imaging without scleral depression but requiring an eyelid speculum in ROP, X-linked retinoschisis, and incontinentia pigmenti, respectively (64). For comparison, the handheld SD-OCT Envisu C2300 used in most studies described in this review has a field of view of 25° (10) while BIO provides a field of view of 69° (23, 65). Achieving consistent peripheral OCT retinal imaging without an eyelid speculum or ocular contact remains a challenge.

## Limitations

Despite significant advancements in handheld OCT devices for the early detection of ROP in research settings, its applicability in clinical settings has not been fully established. Individual OCT biomarkers have not been shown to provide adequate clinical prediction of disease to replace current ROP screening methods. Furthermore, access to OCT imaging is limited in many NICU settings in the United States and internationally due to the cost and challenge of device usability. Most commercially available OCT devices and all non-contact OCT devices have limited ability to visualize the periphery. In general, longer mean examination time for handheld OCT devices (one study found an average time required of 17 min for OCT compared to 5 min for BIO (11) may pose a challenge. Nonetheless, handheld OCT technology is rapidly advancing with potential added clinical value and decreased infant distress compared to existing standard ROP screening methods.

## Conclusions

ROP continues to be one of the leading causes of preventable childhood blindness worldwide. Traditionally, clinical examination with indirect ophthalmoscopy and/or wide-field retinal photography have been the mainstay for ROP screening. The development of a variety of handheld OCT devices has allowed for awake premature infant retinal capture using noncontact, well-tolerated methods for high-resolution cross-sectional vitreoretinal imaging. The use of handheld OCT in a research setting has identified many ROP severity biomarkers coinciding with subclinical structural findings associated with the development and progression of ROP (Table 1). The handheld OCT ROP severity biomarkers discussed in this review have the potential to guide screening and treatment of ROP. Despite this progress, use of handheld OCT for the diagnosis and monitoring of ROP is not yet standard of care in a clinical setting. Quantitative data gathered through handheld OCT devices in conjunction with artificial intelligence or other statistical methods has the potential to identify high risk infants. Further research is needed to validate these approaches on a larger scale.

**Table 1:** Retinopathy of prematurity biomarkers identified by handheld optical coherence tomography.

Biomarkers	Publication(s)	N	Associations
**Vitreous biomarkers**			
Vitreous bands	Zepeda et al. (17) Legocki et al. (12)	65 92	• Cystoid macular edema (*P* = 0.005) • Epiretinal membrane (*P* = 0.001) • Plus disease status (*P* = 0.05)
Punctate hyperreflective vitreous opacities	Legocki et al. (12)	92	• Diagnosis of ROP (*P* = 0.003) • Maximum ROP stage (*P* = 0.001) • *Pre-*plus or plus disease (*P* = 0.005) • Type 1 disease (*P* = 0.03)
Vitreous opacity density	Legocki et al. (12) Scoville et al. (28)	92 78	• Prematurity (*P* = 0.009) • ROP zone (*P* = 0.044) • ROP stage (*P* = 0.031)
**Cystoid macular edema**			
Severity of macular edema	Mangalesh et al. (31) Maldonado et al. (32)	85 62	• Retinal thickness (*P* < 0.001) • Inner nuclear layer (INL) thickness (*P* < 0.001) • Plus disease (*P* = 0.001) • Maximum ROP stage (*P* < 0.001) • Gestational age (*P* = 0.04)
Presence of macular edema	Bondalapati et al. (35) Zepeda et al. (17)	73 65	• Diagnosis of ROP (*P* = 0.03) • Gestational age (*P* = 0.04) • Vitreous bands (*P* = 0.005)
**Foveal development biomarkers**			
Central Foveal thickness	O’ Sullivan et al. (41) Anwar et al. (43)	102 87	• Prematurity (*P* < 0.0001) • Gestational age (*P* < 0.007) • Severity of prematurity (*P* < 0.001)
Parafoveal thickness	Anwar et al. (43)	87	• Gestational age (*P* < 0.007)
Foveal angle	Lawson et al. (42) Anwar et al. (43)	70 87	• Birth weight (*P* = 0.003) • Postmenstrual age (*P* < 0.001) • Gestational age (*P* < 0.001) • Prematurity (*P* < 0.01)
Inner retinal fovea/parafovea ratio	Lawson et al. (42)	70	• Postmenstrual age (*P* < 0.001) • Gestational age (*P* < 0.001) • Birth weight (*P* < 0.001)
Outer retinal fovea/parafovea ratio	Lawson et al. (42)	70	• Gestational age (*P* = 0.002) • Birth weight (*P* = 0.003)
Foveal width	Anwar et al. (43)	87	• Diagnosis of ROP (*P* < 0.001) • Gestational age (*P* < 0.001) • Birth weight (*P* < 0.001)
**Choroidal features**			
Foveal and parafoveal choroidal thickness	Lawson et al. (42)	70	• Postmenstrual age (*P* < 0.001) • Birth weight (*P* < 0.001) • Gestational age (P < 0.001)
Thinner choroid	Mangalesh et al. (31) Erol et al. (34)	85 80	• *Pre*-plus or plus disease (*P* = 0.04) • ROP stage (*P* = 0.01) • Lower gestational age (*P* = 0.01) • Lower birth weight (*P* < 0.001)
**Dome-Shaped Macula**			
Dome-Shaped Macula	Legocki et al. (51)	37	• Diagnosis of ROP (*P* = 0.02) • *Pre*-plus or plus disease (*P* = 0.02) • Birth weight (P = 0.04)
**OCT features of plus disease**			
Vessel elevation	Maldonado et al. (16)	57	• Plus disease (*P* = 0.01)
Hyporeflective vessels	Maldonado et al. (16)	57	• Plus disease (*P* = 0.04)
Scalloped retinal layers	Maldonado et al. (16)	57	• Plus disease (*P* = 0.006)
VASO score	Maldonado et al. (16)	57	• Plus disease (*P* = 0.0013)
**OCT angiography**			
Large vessel tortuosity	Zhou et al. (26)	20	• Prematurity (*P* = 0.004)
Vessel area density	Zhou et al. (26) Vinekar et al. (58)	8 81	• ROP stage (*P* = 0.007) • Pre-plus disease (*P* = 0.007) • Prematurity (*P* = 0.031)
Foveal Avascular Zone (FAZ)	Vinekar et al. (58)	81	• Prematurity (*P* = 0.003) • Gestational age (*P* < 0.035) • Birth weight (*P* = 0.002)
**Peripheral OCT**			
Ridge thickness	Nguyen et al. (62)	25	• ROP stage (*P* < .001)

ROP, retinopathy of prematurity; OCT, optical coherence tomography; VASO, vascular abnormality score by OCT.
